# Effect of Inhibition of DNA Methylation Combined with Task-Specific Training on Chronic Stroke Recovery

**DOI:** 10.3390/ijms19072019

**Published:** 2018-07-11

**Authors:** In-Ae Choi, Cheol Soon Lee, Hahn Young Kim, Dong-Hee Choi, Jongmin Lee

**Affiliations:** 1 Center for Neuroscience Research, Institute of Biomedical Science and Technology, Konkuk University, Seoul 05029, Korea; adia86@naver.com (I.-A.C.); slaoa1428@naver.com (C.S.L.); hykimmd@gmail.com (H.Y.K.); 2 Department of Medical Science Konkuk University School of Medicine, Konkuk University, Seoul 05029, Korea; 3 Department of Rehabilitation Medicine, Konkuk University School of Medicine, Konkuk University, Seoul 05029, Korea

**Keywords:** stroke, chronic stage, task-specific training, DNA methylation, axonal plasticity, functional recovery, mature brain-derived neurotrophic factor

## Abstract

To develop new rehabilitation therapies for chronic stroke, this study examined the effectiveness of task-specific training (TST) and TST combined with DNA methyltransferase inhibitor in chronic stroke recovery. Eight weeks after photothrombotic stroke, 5-Aza-2′-deoxycytidine (5-Aza-dC) infusion was done on the contralesional cortex for four weeks, with and without TST. Functional recovery was assessed using the staircase test, the cylinder test, and the modified neurological severity score (mNSS). Axonal plasticity and expression of brain-derived neurotrophic factor (BDNF) were determined in the contralateral motor cortex. TST and TST combined with 5-Aza-dC significantly improved the skilled reaching ability in the staircase test and ameliorated mNSS scores and cylinder test performance. TST and TST with 5-Aza-dC significantly increased the crossing fibers from the contralesional red nucleus, reticular formation in medullar oblongata, and dorsolateral spinal cord. Mature BDNF was significantly upregulated by TST and TST combined with 5-Azd-dC. Functional recovery after chronic stroke may involve axonal plasticity and increased mature BDNF by modulating DNA methylation in the contralesional cortex. Our results suggest that combined therapy to enhance axonal plasticity based on TST and 5-Aza-dC constitutes a promising approach for promoting the recovery of function in the chronic stage of stroke.

## 1. Introduction

Stroke is the most common cause of long-term adult disability [1]. There is currently no specific treatment for improving functional recovery after stroke, except during rehabilitation. It is well known that rehabilitation plays a pivotal role in the functional motor recovery of patients with stroke [2,3].

Neurological recovery culminates within 1–3 months after stroke. Most spontaneous recovery occurs up to six months after the condition [1,2,3]. The Copenhagen stroke study found that 80% of patients with stroke attain good neurological outcome within 4.5 weeks of stroke, with time profiles varying according to the initial post-stroke severity [4]. Therefore, an appropriate therapeutic period is very important for the restoration of function after stroke. Hence, there is a requirement for the improvement of effective therapeutic options that can enhance functional recovery in chronic stages after stroke.

One such promising therapy is specific behavioral experience (such as motor skill training after experimental brain injury), which provides functional benefits [5,6]. There is emerging evidence of the importance of task-specific training (TST) as a neuromotor intervention in neurological restoration [7,8]. TST can enhance experience-dependent motor skill learning and neural plastic changes in animal and human brain [7,8,9].

In addition, recent findings indicate a positive role of the contralesional hemisphere in the post-stroke recovery of upper extremity motor function [10]. Although motor cortex reorganization of the lesioned hemisphere is involved in post-stroke motor recovery and functions as a target of rehabilitation therapies, reorganization of the motor cortex in the contralateral hemisphere after stroke injury may represent an additional element of cortical reforming and related recovery [10]. Recent reports have demonstrated that the contralesional motor cortex plays an important role in functional recovery after stroke as a potential new target for rehabilitation in human and animal stroke models [5,11,12,13].

Ischemia leads to vast changes in gene expression [14,15,16]. The total levels of DNA methylation increase after ischemic damage and are strongly and directly related to the severity of brain injury [17,18]. Several researchers have expanded their investigations of the role of epigenetic mechanisms in ischemic stroke [19]. Epigenetic mechanisms, i.e., heritable changes in gene expression without changes in the DNA sequence [20], mainly include DNA methylation [14,21], histone modification [22], and microRNAs (miRNAs) [23,24], which specifically modulate the expression levels of single genes and functional gene networks [25,26].

Epigenetics is relevant for cellular response to ischemia. Epigenetic modifiers can influence diverse aspects of this response by altering transcriptional regulation in the ischemic disease process [14]. The transcriptional effects include diminution of cell damage, inhibition of inflammatory reactions, and advanced blood flow, as well as the restoration of mechanisms and enhancement of plasticity [14]. Neural-cell differentiation plasticity is regulated by cell-intrinsic epigenetic mechanisms and ischemic insults in the adult mammalian brain [19]. Furthermore, the targeting of epigenetic mechanisms may lead to the development of new therapeutic approaches for cerebral ischemia [19].

A recent study found that genes and proteins of brain-derived neurotrophic factor (BDNF), proBDNF and their processing enzymes such as tissue plasminogen activator (tPA), furin, and matrix metalloproteinases (MMPs) lead to up-regulation in cerebral ischemia [27]. Thus, this study has suggested that the stability of BDNF and proBDNF and their linked proteins may play a pivotal role in stroke recovery [27].

Therefore, the aims of this study were to implement a novel approach to chronic-phase stroke recovery. This study explored alterations in global DNA methylation from the acute to the chronic phase after stroke in the peri-infarct motor cortex and in the corresponding motor cortex region of the contralateral hemisphere. To develop a beneficial therapeutic protocol at late periods after stroke, task-specific training of the affected forelimb and regulation of DNA methylation were applied to ischemic injured rats. We examined the effects of these treatments on neuronal plasticity, stroke recovery, and neurotrophic factor production. This study will contribute to the development of a promising therapeutic strategy aimed at improving recovery from chronic stroke via TST and the control of DNA methylation in the contralesional cortex.

## 2. Results

### 2.1. Long-Term Post-Stroke Changes, Including Functional Outcome and Brain Injury

**Figure 1:**
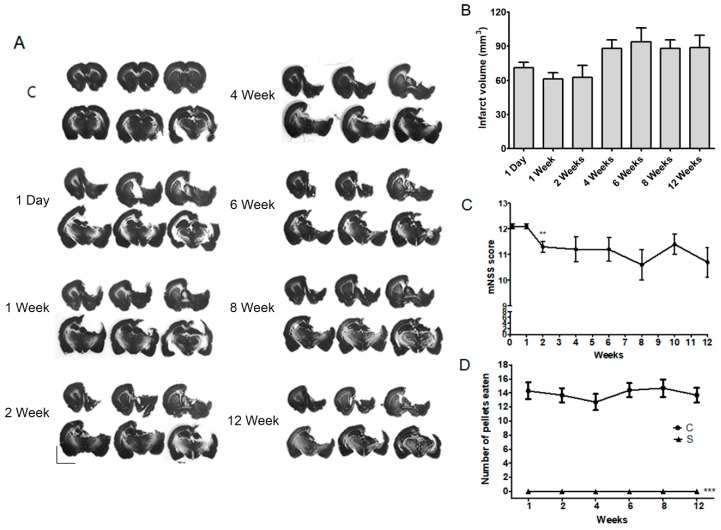
Evaluations of infarct volume and motor function from acute to chronic stages after a photothrombic ischemic stroke: (**A**) Representative photomicrography of Nissl-stained sections at several time points after a stroke; (**B**) Quantification of infarction size did not differ among time points after a photothrombic ischemic stroke (S). Results are presented as the mean ± SEM, *n* = 6; (**C**) while modified NSS levels were slightly improved at 2 weeks after a stroke, after then scores were continued until 12 weeks. Results are presented as the mean ± SEM, *n* = 10. ** *p* < 0.01 vs. 4 days after a stroke; (**D**) motor function impairment of the animals was maintained for 12 weeks after a stroke (S). Results of the staircase test are presented as the mean ± SEM, *n* = 10. *** *p* < 0.001 vs. sham control (C). Scale bars = 5 mm.

To confirm the effectiveness of rehabilitative therapies 2–3 months after a stroke, we assessed changes in infarction volume, motor function, and modified neurological severity score (mNSS) during the early-to-chronic phases after a severe stroke (post-stroke day 1 to week 12). Areas of infarction were noted in the striatum, motor cortex, and sensorimotor cortex. The infarct volumes are shown in Figure 1A. The mean values of the infarct volume in rats that received photothrombotic ischemia were 79.16 ± 5.16 mm^3^ (*n* = 6 per group) at day 1 and weeks 1, 2, 4, 6, 8, and 12 after a stroke. There was no significant difference in infarct volume between the time points (*p* > 0.05, Figure 1B). Motor outcome was evaluated in rats using modified neurological severity score (mNSS). A high score indicated that the rats suffered more neurological defects. Although the mNSS significantly decreased at 2 weeks after the stroke (*p* < 0.01, *n* = 10, Figure 1C), it remained unchanged thereafter (Figure 1C). Staircase tests showed that rats had a significantly impaired functional outcome at 12 weeks after stroke (*p* < 0.001, *n* = 10, Figure 1D). Ischemic injury resulted in a significant reduction in the number of pellets retrieved, when compared with control animals, there was no difference between the time points after stroke (*p* > 0.05, *n* = 10, Figure 1D).

### 2.2. Increase in Contralateral and Ipsilateral DNA Methylation during the Chronic Phase after a Severe Stroke

**Figure 2:**
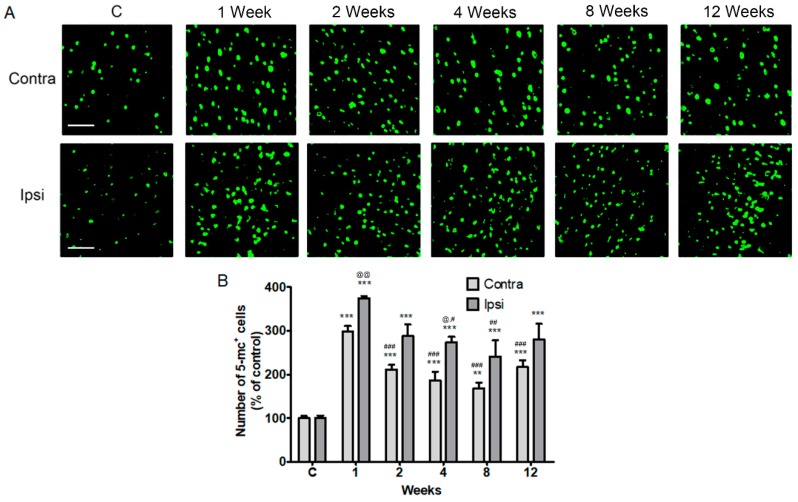
Localization of 5-methylcytosine (5-mc) in the contralesional and ipsilesional cortex after a stroke. (**A**) Fluorescent confocal microscopy shows that the 5-mc (green) is predominantly localized in the both the contralateral (contra) and ipsilateral (Ipsi) cortex from 1 week to 12 weeks after a stroke; (**B**) Quantification of 5-mc levels increased after a photothrombic ischemic stroke. Results are presented as the mean ± SEM, *n* = 6. ** *p* < 0.01; *** *p* < 0.001 vs sham control (**C**); # *p* < 0.05, ## *p* < 0.01, ### *p* < 0.001 vs. 1 day after stroke; @ *p* < 0.05, @@ *p* < 0.01 vs contralateral value at each time. Scale bars = 50 μm.

Next, we confirmed the levels of global DNA methylation detected by 5-methylcytosine (5-mc) in the contralateral and ipsilateral cortices after a stroke. We found that the global DNA methylation levels were significantly increased 1 to 12 weeks (the chronic phase) after a stroke in both the contralateral cortex and the ipsilateral peri-infarct area, compared to the control (*p* < 0.01, *n* = 6, Figure 2). DNA methylation levels peaked at 1 week after a stroke in the contralateral and ipsilateral cerebral cortex. The DNA methylation levels, however, decreased at 2, 4, 8, and 12 weeks in the contralateral cortex and 4 and 8 weeks in the ipsilateral cortex compared with DNA methylation level of 1 week, respectively. The 5-mc level between the ipsilateral and the cortical cerebral cortex after a stroke was different 1 and 4 weeks after a stroke, but not significantly different between 8 and 12 weeks (*p* < 0.05, *n* = 6, Figure 2).

**Figure 3:**
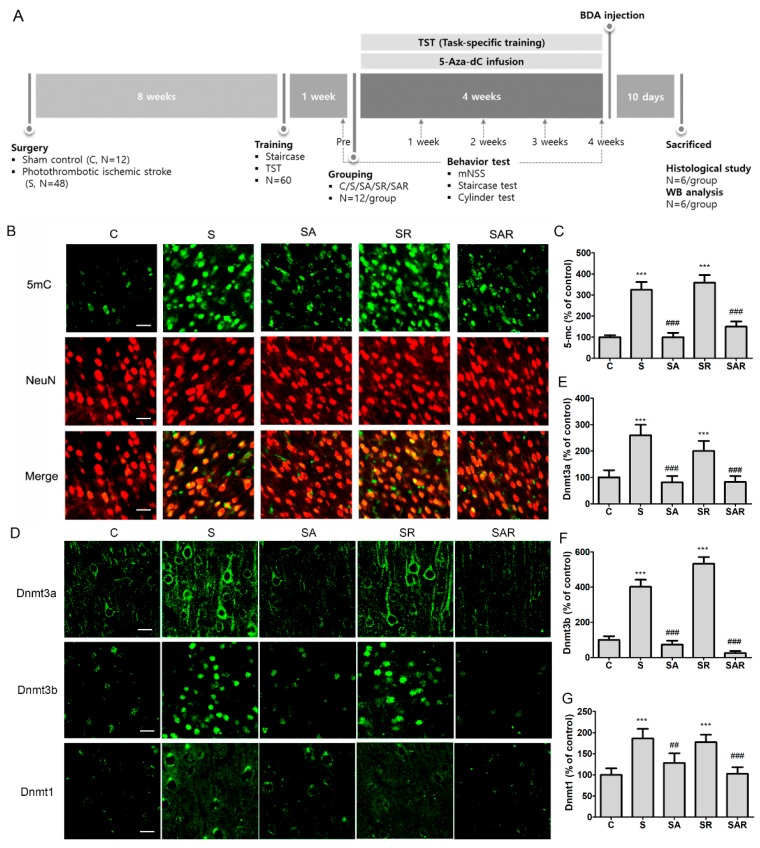
The timeline of the experiment and expressions of 5 mc, Dnmt3a, Dnmt3b, and Dnmt1 in the contralesional cortex after a stroke. (**A**) In a study on the effect of TST and DNMT inhibition on the functional recovery of chronic stroke, sham control (C) and stroke rats (S) with or without TST (SR) and with or without 5-Aza-dC (SA or SAR) were used for behavior test, neural plasticity, protein expression studies, and immunohistochemical analysis; (**B**) representative photomicrograph of 5-mc (green), NeuN (red) and co-localized merged cells (yellow) and (**D**) Dnmt3a, Dnmt3b, or Dnmt1 immunostaining in the contralesional cortex after stroke; (**C**,**E**–**G**) Counts of 5-mc, Dnmt3a, Dnmt3b, or Dnmt1 positive cells. Results are presented as the mean ± SEM, *n* = 6/group. *** *p* < 0.001 vs. C, ## *p* < 0.01; ### *p* < 0.001 vs. S, Scale bars = 20 μm.

We hypothesized that regulation of contralateral DNA methylation levels and TST contributes to motor recovery in the chronic phase after a stroke. To assess the effects of TST and DNA methyltransferase (DNMT) inhibitor 5-Aza-2′-deoxycytidine (5-Aza-dC), we treated the contralesional cortex of rats, carrying a photothrombotic ischemic stroke unilateral lesion eight weeks after injury, with TST. A detailed timeline for the experiment is provided in Figure 3A. To address this hypothesis, we first confirmed the presence of changes in the levels of DNA methylation and in DNMT1, DNMT3a, and DNMT3b expression after 5-Aza-dC treatment of the contralateral cortex after a stroke. We found that the increased levels of 5-mc in neurons in the contralateral cortices of a stroke control group (S) and TST-treated stroke group (SR) decreased on 5-Aza-dC administration in 5-Aza-dC treated stroke group (SA) and TST and 5-AzadC treated stroke group (SAR) (*p* < 0.001, *n* = 6, Figure 3B,C). The levels of DNMT3a, DNMT3b, and DNMT1 in the contralesional cortex after a stroke (*p* < 0.001, *n* = 6, Figure 3D–G) significantly increased. These increased levels were downregulated in the SA and SAR groups (*p* < 0.05, *n* = 6, Figure 3D–G). 

### 2.3. Infarct Volume Was Not Altered by TST with or without 5-Aza-dc in the Chronic Phase after Severe Stroke

**Figure 4:**
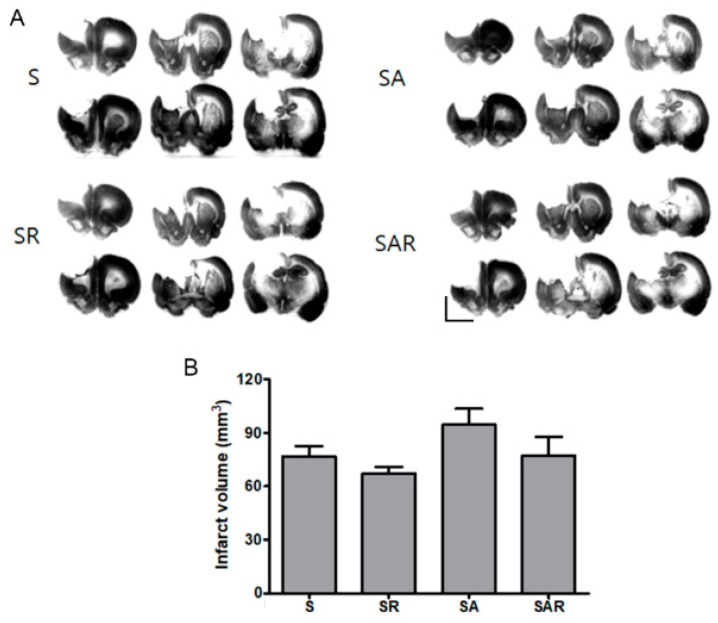
Effects of TST and 5-Aza-dC treatment on infarct volume after photothrombic ischemic stroke (**A**) Representative photomicrography of Nissl-stained sections four weeks after post-stroke treatment; (**B**) Quantification of infarction size did not differ in treatment groups after photothrombic ischemic stroke. Results are presented as the mean ± SEM, *n* = 6. Scale bars = 5 mm.

To determine the effects of TST and 5-Aza-dC on ischemic injury, we examined infarction volume using Nissl staining. Infarct volume in the SR, SA, and SAR groups did not differ from that observed in the stroke control group (S) (*p* = 0.895, *n* = 6, Figure 4A,B).

### 2.4. 5-Aza-dC Combined with TST Treatment Improved the Recovery of Motor Function on the Chronic Phase after Severe Stroke

**Figure 5:**
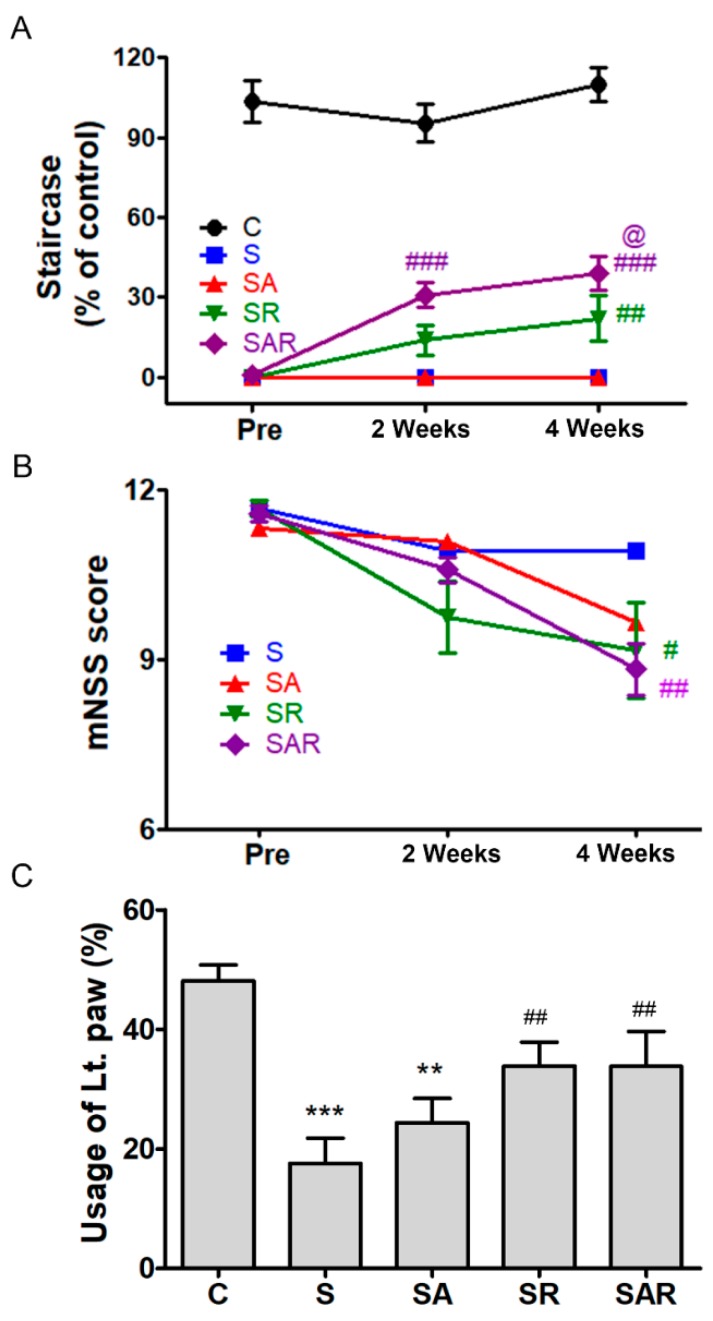
Recovery of motor function on treatment with TST and 5-Azd-dC after a photothrombotic chronic stroke. TST and TST combined with 5-Aza-dC treatment helped improve recovery of motor function after stroke; the staircase test (**A**) and mNSS (**B**), usage scores of damaged limb (**C**). TST combined with 5-Aza-dC enhanced motor recovery in the treated group, as shown in the staircase test performance (**A**). Pre-indicated 1 day before rehabilitation after a stroke. ** *p* < 0.01; *** *p* < 0.001 vs. C (sham control), # *p* < 0.05; ## *p* < 0.01, ### *p* < 0.001 vs. S (Stroke). @ *p* < 0.05 vs. SR (TST treated stroke). Results are presented as the mean ± SEM, *n* = 12/group. Lt. paw: impaired left paw.

To determine the effects of TST and 5-Aza-dC on functional recovery after a chronic stroke, we examined motor recovery and neurological function using the Montoya staircase test, mNSS, and cylinder test (Figure 5).

#### 2.4.1. Montoya Staircase Test

The Montoya staircase test is used to assess the recovery of reaching and grasping skills, impaired by severe stroke. The lab animals were given 18 food pellets, and the number of pellets consumed was determined. Figure 5A shows the results of successful reaches throughout the course of the experiment. The reaching success was measured as the number of pellets retrieved and eaten by the paw contralateral to the lesion. A repeated-measures two-way ANOVA detected significant effects of group (F4,55 = 140.8; *p* < 0.001) and time (F2,55 = 17.45; *p* < 0.001), and a significant interaction between group and time (F8,112 = 7.097; *p* < 0.001). The stroke control group (S) exhibited profound impairment in reaching success (*p* < 0.001, *n* = 12, Figure 5A). The SR group, which received TST after stroke, and the SAR group, which received TST and 5-Aza-dC after stroke, exhibited significant improvement, compared to the S group (*p* < 0.001, *n* = 12, Figure 5A). The SAR group exhibited a more-pronounced improvement in performance compared to the SR group (*p* < 0.01, *n* = 12, Figure 5A). No differences were observed between the S and the SA group, which only received 5-Aza-dC.

#### 2.4.2. mNSS

The SR and SAR groups exhibited significant amelioration in mNSS, compared to the S group at four weeks after a stroke. A repeated-measures two-way ANOVA detected significant effects of time (F3,44 = 33.38; *p* < 0.001) and interaction between group and time (F6,88 = 2.944; *p* < 0.05). At four weeks after a stroke, there was a pronounced improvement in the groups that underwent TST (SR) and TST combined with 5-Aza-dC (SAR) (*p* < 0.05, *n* = 12) (Figure 5B).

#### 2.4.3. Cylinder Test

The decreased usage of the impaired paw ameliorated in the SR and SAR groups at four weeks after a stroke (Figure 5C). ANOVA detected a significant main effect of group (F4,55 = 9.228; *p* < 0.001).

### 2.5. Aza-dC Treatment with TST Enhanced the Neuronal Plasticity of Motor Pathways

**Figure 6:**
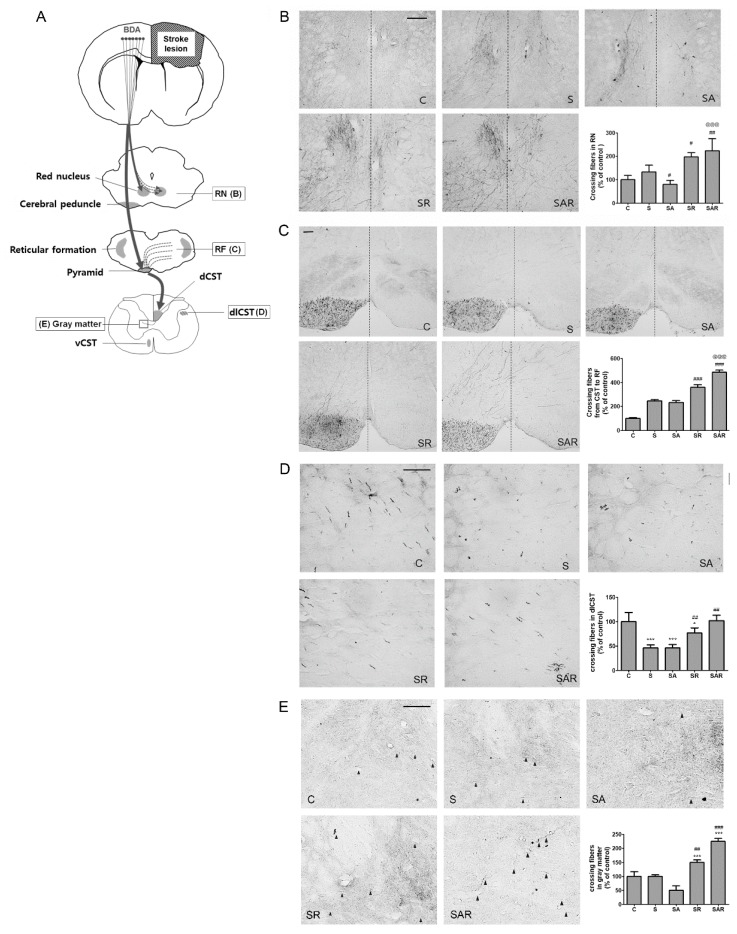
Task-specific training (TST) and TST combined with 5-Aza-C treatment promote contralesional CST plasticity in chronic stroke. (**A**) Schematic illustration of CST including contralesional red nucleus (RN), reticular formation (RF) in medullar oblongata, dorsolateral spinal tract (dlCST) and ipsilesional gray matter in the spinal cord. Solid square boxes indicate analysis area for CST plasticity in this study; (**B**–**E**) photomicrographs of BDA staining in the contralesional RN, RF in medullar oblongata, dlCST and ipsilesional gray matter in spinal cord sections. (**B**,**C**) Broken lines in RN and RF indicate the midline of the midbrain; (**E**) midline-crossing fibers indicate black arrowheads. dCST: dorsal CST; dlCST: dorsolateral CST; vCST: ventral CST. Scale bars = 50 μm. Mean density of BDA-positive axonal crossing fibers in the RN (**B**), RF (**C**), dlCST (**D**), and gray matter of spinal cord (**E**) shown as% increase of sham control (**C**). Results are presented as the mean ± SEM in the graphs. *n* = 6. Significance is indicated by * *p* < 0.05; *** *p* < 0.001 vs. sham control (C), # *p* < 0.05; ## *p*<0.01; ### *p* < 0.001 vs. Stroke (S), @@@ *p* < 0.001 vs. SR.

The corticospinal tract (CST) was labeled with biotinylated dextran amine tracer (BDA) via injection into the contralesional side of the motor cortex, and labeled CST axon fibers sprouting from descending fibers in the non-ischemic side of the red nucleus (RN), medullary reticular formation (RF), pyramid, and cervical spinal cord were examined [28]. The location of the BDA injections produced no major differences between groups and the density of CST fibers showed no differences between groups before stroke in our previous study [5]. First, we examined whether TST and 5-Aza-dC treatment in stroke rats influenced the formation of CST fibers. Tracing of the CST revealed that the number of crossing fibers from the contralesional RN was significantly increased in the SR and SAR groups (Figure 6B). The quantitative data showed the presence of significant increases in the mean number of crossing fibers in the RN of the SR and SAR groups after stroke, by 251.32% ± 8.37% and 288.93% ± 24.69% compared with the stroke (S) group, respectively (*p* < 0.05, *n* = 6, Figure 6B); in contrast, significant decrease was observed in the SA group after stroke (*p* < 0.05, *n* = 6, Figure 6B). The SAR group showed an enhancement in the number of crossing fibers compared with the SR group (*p* < 0.001, *n* = 6, Figure 6B). Next, we quantified the number of BDA-labeled CST fibers that crossed the midline toward the ischemic side in the RF (Figure 6C). The crossing fibers from the pyramidal to RF in the medulla were increased. Regarding the crossing fibers, a statistical analysis using one-way ANOVA revealed that BDA-labeled fibers significantly increased after treatment with TST and 5-Aza-dC combined with TST, by 146.18% ± 9.20% and 197.84% ± 7.45% compared with the stroke (S) group, respectively (*p* < 0.001, *n* = 6, Figure 6C). The number of these fibers in the SAR group was significantly higher than that observed in the SR group after stroke (*p* < 0.001, *n* = 6, Figure 6C). The fibers in the dorsolateral parts of the spinal cord (dlCST) was increased in the SR and SAR group after stroke, by 166.68% ± 9.30% and 221.68% ± 9.83% compared with the stroke (S) group, respectively (*p* < 0.01, *n* = 6, Figure 6D). The CST fibers in ipsilateral gray matter that came from dorsal CST were enhanced in the SR and SAR groups, by 150.60% ± 16.22% and 225.08% ± 9.78% compared with the stroke (S) group, respectively (*p* < 0.01, *n* = 6, Figure 6E).

### 2.6. The Level of Mature Brain-Derived Neurotrophic Factor (BDNF) in the Contralateral Cortex Was Increased by 5-Aza-dc Combined with TST Treatment

**Figure 7:**
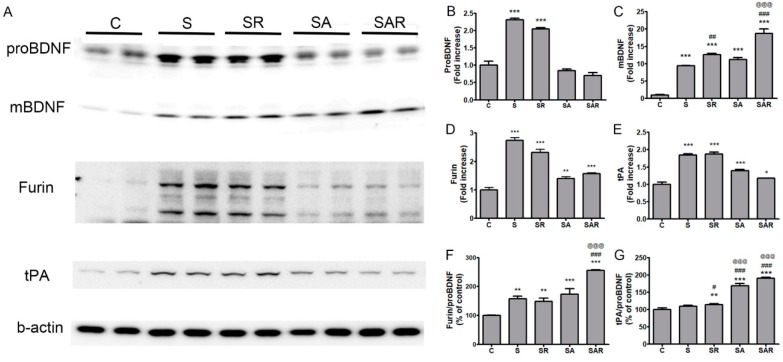
Effect of TST and TST combined with 5-Aza-dC treatment on the expression of mature BDNF in protein extracts of contralesional cortex after stroke. (**A**) Representative photomicrographs of western blots for pro-brain-derived neurotrophic factor (proBDNF), mature BDNF (mBDNF), furin, tissue plasminogen activator (tPA), and β-actin in total lysates of the contralesional cortex at 4 w after post-stroke treatment. (**B**–**E**) Signal intensities of proBDNF, mBDNF, furin and tPA were measured using Quantity One software and are shown as a percentage of control. (**F**,**G**) Signal intensities of the ratio of furin to proBDNF and tPA to proBDNF are shown as a percentage of control. Beta-actin, internal control. Results are presented as the mean ± SE, *n* = 6. * *p* < 0.05; ** *p* < 0.01; *** *p* < 0.001 vs. C, # *p* < 0.05; ## *p* < 0.01; ### *p* < 0.001 vs. S, @@@ *p* < 0.001 vs. SR.

To determine whether the expression and maturation of BDNF were affected by TST and 5-Aza-dC treatment and enhanced contralateral neuronal plasticity after stroke, we immunoblotted protein extracts from contralesional cortical tissues. The pro-brain-derived neurotrophic factor (proBDNF) levels in the contralesional side were increased in the S and SR groups compared with the control (*p* < 0.001, Figure 7A,B). However, mature BDNF (mBDNF) levels were higher in the S, SR, SA, and SAR groups compared with the control (*p* < 0.001, *n* = 6, Figure 7A,C). The SR and SAR groups exhibited an enhancement in mBDNF expression compared with the S group (Figure 7A,C). Mature BDNF levels were significantly higher in the SAR vs. the SR group (Figure 7A,C). Next, we examined the effects of TST and 5-Aza-dC on the expression of intracellular (furin) and extracellular tissue plasminogen activator (tPA) proteases that are involved in proBDNF processing in the brain. Furin and tPA expression in the contralesional cortex was significantly increased in the S, SR, SA, and SAR groups (*p* < 0.05, *n* = 6, Figure 7A,D,E) after stroke. We also measured and compared the ratios of furin or tPA and proBDNF among the groups. The ratio of furin to proBDNF was significantly increased in the S, SR, SA, and SAR groups. The ratio observed in the SAR group was higher than that detected in the S or SR groups (*p* < 0.001, *n* = 6, Figure 7F). The ratio of tPA to proBDNF was higher in the SR, SA, and SAR groups compared to the control group. The SA and SAR groups exhibited a significant increase in the ratio vs the SR group (*p* < 0.01; *p* < 0.001, *n* = 6, Figure 7G).

## 3. Discussion

In the present study, we confirmed the effects of TST and inhibition of contralesional DNA methylation on functional outcome, neuronal plasticity, and expression of axonal-growth-enhancing molecules (such as BDNF) in the chronic stage after stroke in a rat model. Although the beneficial effects of TST with affected forelimb and inhibition of ipsilateral DNA methylation have been suggested for the acute stage of stroke, it is uncertain whether TST and the regulation of contralesional DNA methylation have any positive effects on the chronic stage of ischemic stroke. Several studies have demonstrated that rehabilitative training, such as TST, plays beneficial roles in improving motor performance at the acute or subacute stage after stroke [5,7,8,29]. Roles of epigenetics in cerebral ischemia have also been reported, as inhibiting histone deacetylase 2 (HDAC2) promotes functional recovery [30,31] and protection against ischemic insult by inhibiting DNA methylation after treatment with a DNA methyltransferase (DNMT) inhibitor, 5-aza-2′-deoxycytidine (5-Aza-dC) [17,18,20].

Our results demonstrated that TST and TST combined with 5-Aza-dC treatment enhanced behavioral performance, as assessed using the modified neurological severity score, the staircase test, and the cylinder test. In particular, significant differences were observed in the staircase test after stroke between the TST-treated group and the TST combined with 5-Aza-dC-treated group. These findings suggest that motor recovery is enhanced to a greater extent by the inhibition of DNA methylation combined with TST than it is by TST alone. Therefore, epigenetic regulation after stroke may enhance improvement in motor function provided by TST at the chronic stage.

Neuronal network reorganization is a major mechanism that is used to maintain neuronal functions after brain injury [28]. The reorganization of the neural network after a stroke leads to recovery from functional deficits in the remaining areas [32,33]. The motor cortex of the undamaged side may have a supplementary function that restores lost motor functions [33,34].

Axonal sprouting occurs mainly after stroke in the peri-infarct cortex, near the site of stroke. Furthermore, axonal sprouting appears to arise from the contralateral cortex to the ipsilateral RN and the cervical spinal cord [33,35,36]. After severe strokes, plastic alterations are observed from the unaffected cortex to subcortical efferent projections of the corticospinal tract (CST) [33,35]. New CST axons sprout into the lesional subcortical areas at multiple levels of the brain and spinal cord [37]. The process of enhanced axonal sprouting involves axonal growth and promotes functional recovery after stroke [33,37].

TST is normally used in patients with chronic stroke [7]. Axonal remodeling is accepted as one of the components of TST-induced functional recovery and has been detected in parts of the CST [7]. Several studies of the beneficial effect of TST on functional recovery after injury have reported that rehabilitative training induces the growth of saved CST axons and the projection of new axons into the damaged spinal cord, subsequently contributing to motor recovery [8,29,38,39]. Our previous research demonstrated that early TST after stroke enhanced the contralesional plasticity of the CSTs in motor cortical and sensorimotor cortical lesions at the acute stage after stroke [5]. A recent report showed that the number of CST axonal fibers sprouting from the non-ischemic hemisphere was increased in the ipsilateral medullary reticular formation and cervical spinal cord after middle cerebral artery occlusion in rats [28].

In this study, the data showed that the number of crossing fibers from the unaffected side to the RN and medullary reticular formation in the affected side was significantly increased by TST and TST combined with 5-Aza-dC treatment after stroke. The number of spinal cord fibers located in the dlCST and the number of crossing fibers in the gray matter was increased by TST combined with 5-Aza-dC treatment after stroke. Taken together with previous reports, our data suggest that regulation of DNA methylation in the contralesional cortex positively contributes to the enhancement of neuronal plasticity afforded by TST of the affected forelimb in motor recovery at the chronic phase after stroke.

Post-stroke motor recovery involves regaining and relearning skills and is related to neural plasticity [40]. Although various molecular signaling pathways are engaged in neural plasticity and recovery after stroke, BDNF signaling has emerged as a key player in these processes [40]. The activity-dependent upregulation of BDNF contributes to the minimization of the extent of cell death during both the acute phase (hours to days) and the subacute phase (days to weeks), thus promoting plasticity and improving function [41]. Previous reports have demonstrated that exercise [40,42,43], environment enrichment [44,45], and neuronal pharmacotherapy [46,47,48] after stroke mediate neuroplastic changes through the expression of BDNF in the contralateral and ipsilateral hemispheres [49]. Cerebral ischemia in rats led to the upregulation of the BDNF precursor protein (proBDNF), mature BDNF (mBDNF), and their processing enzymes, such as furin and prohormone convertases, in the intracellular milieu, and matrix metalloproteinases (MMPs) or plasmin in the extracellular milieu [27,50]. The mBDNF protein (converted from proBDNF) is mainly involved in the promotion of functional recovery after ischemia [27,51,52,53].

Our data showed that the expression of mBDNF was significantly increased by TST and TST combined with 5-Aza-dC treatment after stroke. The expression of proBDNF-converting enzymes, such as tPA and furin, was also increased by TST combined with 5-Aza-dC treatment. These results suggest that the inhibition of DNA methylation by 5-Aza-dC treatment might alter the production of mBDNF via the regulation of the expression of tPA or furin.

On the other hand, 5-Aza-dC treatment alone after stroke did not show protective effects. Effect of 5-Aza-dC may be different between control-contralateral cortex and TST-treated contralateral cortex. TST-induced microenvironmental status after stroke may differ from stroke without TST. 5-Aza-dC treatment in the contralateral cortex after an ischemic stroke may provide a supportive microenvironment for the repairing process promoted by TST [54].

Therefore, the overall data generated in this study suggest that TST with an affected forelimb at the chronic phase after severe stroke plays a beneficial role in functional recovery via neuronal plastic changes, enhanced proBDNF expression, and mBDNF production in the contralesional cortex. Those effects were significantly enhanced by additional inhibition of DNA methylation in the contralateral motor cortex after a stroke, and were mediated by increased CST plasticity and mBDNF products (via the upregulation of tPA and furin enzymes).

## 4. Materials and Methods

### 4.1. Animals

A total of 142 male Wistar rats (8 weeks of age, weighing 283.88 ± 2.06 g; Orent Bio Inc., Seongnam, Korea; 24 sham control rats (C) and 118 photothrombotic-stroke rats, Stroke) were used. The study animals were exposed to a temperature-controlled room (23 ± 0.5 °C) with 12-h light/dark cycle. All experimental procedures were approved by the Animal Experiment Review Board of Institutional Animal Care and Use Committee (IACUC) of Konkuk University (Permit Number: KU17042, licensed on 21 March 2017). Animal care, including anesthesia and euthanasia, was performed in accordance with the Principle of Laboratory Animal Care (NIH publication No. 85-23, revised 1985). The authors also followed the criteria for Stroke Therapy Academic Industry Roundtable (STAIR) for preclinical stroke investigations [55].

### 4.2. Photothrombotic Ischemia Surgery

The cerebral cortical infarct was made by projecting light onto the sensory motor and motor cortex after Rose Bengal treatment. To briefly summarize the method, the rats were anesthetized with a mixture of ketamine (50 mg/kg) and xylazine (5 mg/kg) After intraperitoneal (ip) injection, the animals were anesthetized and placed in a fixed bed frame (Stoelting Co., Wood Dale, IL, USA). The skull was exposed and a fiber bundle of 4 mm caliber KL1500 LCD cold light source (Carl Zeiss, Jena, Germany) was placed in the bregma of the skull and placed 4.0 mm lateral to the midline of the right sensory motor. Photochemical dye Rose Bengal (Sigma-Aldrich, St. Louis, MO, USA) was infused via i.p. injection. After injection for 5 min (20 mg/kg), the light was switched on for 30 min. Sham control animals were exposed to light for 30 min without the injection of Rose Bengal.

### 4.3. Animal Grouping

In the temporal change study conducted on 80 rats, 70 underwent photothrombotic ischemic stroke surgery and 70 survivors were equally distributed to 1 day, 1 week, 2, 4, 6, 8, and 12 weeks after stroke (*n* = 10 rats/group). Ten sham control rats were treated similarly to the operated rats, except for exposure to light source. In the rehabilitation therapy study, 48 rats were subjected to photothrombic stroke. After 4 weeks, 24 rats were injected with 5-Aza-dC (10 μg/day, Sigma-Aldrich, St. Louis, MO, USA) using osmotic minipump. After 3 days, 12 rats of the 24 stroke rats and 12 rats of 5-Aza-dC treated stroke rats were provided task-specific training (TST). Twelve stroke rats and 12 sham control rats were not treated with 5-Azd-dC or TST. All experimental groups were randomly allocated to the following treatment groups: sham control (C, *n* = 12), stroke control (S, *n* = 12), TST-treated stroke rats (SR, *n* = 12), 5-Aza-d-C treated stroke rats (SA, *n* = 12), TST and 5-Aza-dC cotreated stroke rats (SAR, *n* = 12). Animals were number-coded and investigators were blinded to the treatment groups until the end of data analysis. 

### 4.4. Infusion of 5-Aza-dC in Contralateral Motor Cortex

5-Aza-dC (10 μg/day) was delivered to the contralesional hemisphere sensorimotor cortex using osmotic pump system for 28 days. Osmotic minipumps (0.25 μg/h, 200 μL volume, model 2004, Alzet, Cupertino, CA, USA) filled with 10 mg 5-Aza-dC in 3% DMSO solution 200 μL placed into 15 mL conical tubes containing 0.9% saline were primed at 37 °C incubator 2 days before the implantation surgery. Animals were anesthetized with ketamine (50 mg/kg) and xylazine (5 mg/kg) mixed cocktail through intraperitoneal (i.p.) injection then their head were fixed at a stereotaxic frame (Stoelting Co., Wood Dale, IL, USA). The skull was exposed and the cannula (Brain infusion kit3, Alzet, Cupertino, CA, USA), which is connected to osmotic pump through tube, was implanted on the sensorimotor cortex of the contralesional hemisphere (ML 2, AP 0.5, DV-2). Screws (2 mm diameter) were anchored onto the skull to secure the implantation; dental cement (Poly-F standard kit, Dentsply, York, PA, USA) then covered the skull. Osmotic minipumps were placed subcutaneously on the back of each rat.

### 4.5. Task-Specific Training

Nine weeks after induction of photo-thrombotic stroke, animals were implanted with osmotic pump delivering 5-Aza-dC. It took 3 days to recover from the surgery; rehabilitation training then begun. Animals were randomized to the following treatment groups: sham control (C, *n* = 12), stroke control (S, *n* = 12), stroke with task-specific training (TST) (SR, *n* = 12), stroke with 5-aza-d-C (SA, *n* = 12), stroke with TST combined with 5-aza-dC (SAR, *n* = 12). The task-specific training was performed as described previously for 4 weeks [55].

### 4.6. Staircase Test

The training began from 8 weeks after surgery and the animals received daily handling for 1 week. They were feed restricted for 2 weeks during staircase training (two trials per day, 15 min each) and their weight was checked every day; body weight was not under 85% of start weight. Animals were trained to reach for feed pellets in the modified staircase apparatus (three pellets per step with 6 steps on each side) according to previously published research [55]. The total number of pellets eaten was counted. The performance score was calculated using the following formula: the number of pellets eaten/total number of pellets.

### 4.7. Modified Neurological Severity Score

Animals were examined with modified neurological severity score (mNSS) one day prior to rehabilitation, and 1, 2, 3 and 4 weeks after rehabilitation. This evaluation was performed by a blinded tester. mNSS consists of motor, sensory, reflex and balance test. Scores ranged from 0 to maximum 14. mNSS was performed as described previously. 

### 4.8. Cylinder Test (Asymmetrical Forelimb Use)

The animals were placed in a transparent Plexiglas cylinder (diameter 20 mm) placed on a glass plate and videotaped with a camcorder from below. Single (ipsilateral and contralateral) and bilateral forelimb wall contacts were recorded for 5 min (or until more than 15 wall contacts were observed). Contralateral forelimb use was expressed as follows: ([contralateral forelimb contacts + 0.5 × bilateral forelimb contacts]/total number of forelimb contacts) × 100 [5]. Animals were tested several times before and after recovery therapy.

### 4.9. Video Recording

Filming was performed 1 day before rehabilitation after photothrombotic ischemia, and 1, 2, 3, and 4 weeks of training or 5-Aza-dC treatment using a Sony HDR-CX350 Handycam (Tokyo, Japan). The animals were filmed from frontal and ventral viewpoints. The tapes were viewed on a Sony DV cam HDR-CX350 player. Representative movements were captured by GOM Player v2.3 (Seoul, South Korea) using a Windows 10 computer.

### 4.10. Nissle Staining

The rats (*n* = 6/group) were deeply anesthetized with a mixture of ketamine (50 mg/kg) and xylazine (5 mg/kg) (ip) followed by saline containing 0.5% sodium nitrite and 10 U/mL heparin sulfate. After perfusion, the brains were perfused with cold fixative solution of 0.1 M PBS (pH 7.2) containing 4% formaldehyde, and the brains were postfixed overnight in the same solution and infiltrated with 30% sucrose. Using a cryostat, a floating section (40 μm) from bregma −5.2 to 2.2 mm was obtained and the section was mounted on a glass slide and stained with Nissl [56]. 

### 4.11. Measurement of Infarct Volume

Using Nissl stained sections, the infarct volume (total cortex and posterior cortex) was quantified using image analysis software (Image J v1.3 (Bethesda, MD, USA), NIH) to calibrate cerebral edema according to the following formula: CIV = (LHA − RHA − RIA) × Thickness, CIV is the corrected infarct volume, LHA is the left hemisphere, RHA is the right hemisphere, RIA is the right hemisphere infarct area. The total infarct size was estimated using the corrected infarct area and the width between individual brain slices [56].

### 4.12. CST Projections Using Biotinylated Dextran Amine

The anterograde tract tracer biotinylated dextran amine (BDA) was used to evaluate pyramidal tract plasticity contralateral of stroke in rats subjected to permanent focal cerebral ischemia [55]. One percent of BDA was stereotaxically injected into the motor cortex at 4 weeks after post-rehabilitation treatment in photothrombotic ischemic rats (*n* = 6/group). Ten days after BDA injection (at 4 weeks after rehabilitation), the rats were deeply anesthetized (ketamine and xylazine mixture 30 mg/kg, i.p.) and placed in a rat stereotaxic apparatus. BDA was then injected into contralateral site in the motor cortex (coordinate: AP, −1.5, 0, 0.5, 1, 1.5, 2, 2.5 mm; ML 1.5, 2.0, 1.8, 2.5, 2.5, 2.5, 3 mm; dorsoventral (DV), −1.5 mm). Each injection of BDA in 1 μL ice-cold sterilized phosphate buffered saline was used in every animal. The injection rate was 0.2 μL/min, and the syringe was kept in place for an additional 5 min before being retracted slowly. Thus, BDA was visualized by immunohistostaining. 

### 4.13. Immunohistochemistry

Forty-μm-thick coronal cryosections of the brain were selected, each comprising six sections, including the red nucleus (RN) and pyramid (−8.72 to −11.60 mm at AP), and the spinal cord (SP) to detect BDA labeling. Briefly, free-floating sections were incubated with 0.3% H_2_O_2_ in 0.1 M phosphate-buffered saline (PBS, pH 7.4) for 20 min, washed with 0.1 M PBS, then incubated in 0.1 M PBS containing 5% normal horse serum and 0.3% Triton X-100 for 1 h. The sections were incubated with avidin–biotin–peroxidase complex (Vector Laboratories, Burlingame, CA, USA) in PBS/Triton X-100 at 4 °C for 3 days and BDA labeling was achieved with 0.05% 3,3′-diaminobenzidine and 0.003% H_2_O_2_ (Vector Laboratories) before light microscopy examination [5].

### 4.14. Western Blot Analysis

The contralesional cortices washed with PBS was lysed with RIPA buffer (50 mM Tris-HCl pH 7.4, 150 mM NaCl, 1% NP40, 0.25% Na-deoxycholate, and 0.1% SDS) containing a protease inhibitor mixture and phosphatase inhibitors (Sigma-Aldrich, St. Louis, MO, USA). (BDNF, tPA, furin: Santa Cruz (R)) was added to SDS-PAGE and electroporated into Polyvinylidene fluoride membrane. Thirty micrograms of soluble protein were subjected to SDS–PAGE and electrotransferred onto a PVDF membrane. Specific protein bands were detected using specific antibodies (BDNF, tPA, furin: Santa Cruz biotechnology, Inc., Dallas, TX, USA, and β-actin: Sigma-Aldrich, St. Louis, MO, USA) and enhanced chemiluminescence (Pierce, Rockford, IL, USA) [55].

### 4.15. Double-Fluorescence Immunostaining of Tissues

Free-floating sections (40 µm) were incubated in 0.1 M PBS containing 5% normal donkey serum and 0.3% Triton X-100 for 1 h, and subsequently incubated overnight with specific primary antibodies (NeuN: Millipore, Burlington, MA, USA, 5-mc: Active Motif, Carlsbad, CA, USA, Dnmt3a, Dnmt3b, and Dnmt 1: Santa Cruz biotechnology, Inc., Dallas, TX, USA) in 2% normal donkey serum (Vector Laboratories, Burlingame, CA, USA) in PBS at 4 °C and incubated with a 1:200 dilution of Alexa Fluor-conjugated donkey anti-rabbit (488) or donkey anti-mouse (546) antibodies (Invitrogen, Grand Island, NY, USA) for 1 h at room temperature and mounted on glass slides using Vectashield (Vector Laboratories, Burlingame, CA, USA). Fluorescent signals were evaluated on a confocal microscope (LSM 710, Carl Zeiss, Oberkochen, Germany) [55].

### 4.16. Quantitative Analysis

The cortex sections from 6 rats per group were subjected to analysis. Five regions of interest (ROIs) of 0.1 mm^2^ per one section were selected. The number of NeuN, 5 mc, DNMT1, DNMT3a, and DNMT3b-positive cells was counted in each ROI and averaged. Data are represented as the percentage of total cells. All quantitative analyses were carried out in a blind manner [55].

### 4.17. Analysis of CST Projections

Images were captured and analyzed with Axio Vision using a CCD camera (Jena, Germany) attached to an inverted light microscope with 10× or 20× objectives (Carl Zeiss, Jena, Germany). The mean account of the axonal fibers was quantified by an observer who was blind to the grouping using an automated program wizard, i.e., the “measurement” plug-in of Axio Vision. Objects of interest (fibers) were selected using the segmentation command. Artifacts were deleted manually from the selected group of objects. The number of crossing axonal fiber (μm^2^) was measured based on the total area of a captured image. Five captured images were analyzed for each group.

### 4.18. Data Analysis and Statistics

The staircase test and mNSS was analyzed using a two-way repeated-measures analysis of variance (ANOVA), followed by a post hoc least significant differences multiple comparisons test. A one-way ANOVA was used to compare the infarct volume, cylinder test, intensity of Western blot results, cell counts after immunostaining, and counts of crossing fibers among groups. This statistical analysis comprised a one-way ANOVA followed by a Newman–Keuls multiple comparisons test. All data were expressed as the mean ± standard error. Null hypotheses of no differences were rejected if *p* < 0.05. All data analyses were performed using the SPSS version 22.0 software (IBM Corporation, New York, NY, USA).

## 5. Conclusions

This study demonstrated the beneficial role of TST in motor recovery in the chronic stage after stroke. Moreover, it showed that inhibition of DNA methylation in the contralesional cortex combined with TST enhanced motor function. The combination of contralesional inhibition of DNA methylation and TST with an affected limb may be especially effective for improving motor function after stroke, even if the initiation of rehabilitation occurs during a late phase. Enhanced axonal plasticity in the contralesional corticospinal tract, including the RN, pyramid, and spinal cord, is involved in motor recovery. The molecular mechanism underlying this axonal remodeling may rely on mature BDNF production induced by TST combined with inhibition of DNA methylation after stroke. Further studies are needed to elucidate the changes in molecule-specific DNA methylation induced by treatment with 5-Aza-dC in the contralateral motor cortex after stroke. Therefore, combined therapy of TST and 5-Aza-dC after stroke may constitute a promising therapy for promoting the recovery of function in the chronic stage of stroke.

## Abbreviations

TST	Task specific training
DNMTs	DNA methyltransferases
5-mc	5-methylcytosine
mNSS	Modified neurological severity score
5-Aza-dC	5-aza-2′-deoxycytidine
C	Sham Control group
S	Stroke group
SA	5-Aza-dC treated stroke group
SR	TST treated stroke group
SAR	5-Aza-dC combined with TST treated stroke group
ANOVA	Analysis of variance
RN	Red nucleus
RF	Reticular formation
CST	Corticospinal tract
BDA	Biotinylated dextran amine
dlCST	Dorsolateral parts of the spinal cord
Pro-BDNF	Pro-brain-derived neurotrophic factor
mBDNF	Mature BDNF
t-PA	Tissue plasminogen activator
